# Effect of prolonged HAART on oral colonization with *Candida *and candidiasis

**DOI:** 10.1186/1471-2334-6-8

**Published:** 2006-01-20

**Authors:** Yun-Liang Yang, Hsiu-Jung Lo, Chien-Ching Hung, Yichun Li

**Affiliations:** 1 Department of Biological Science and Technology, National Chiao Tung University, Hsinchu, Taiwan, R.O.C; 2 Division of Clinical Research, National Health Research Institutes, Miaoli, Taiwan, R.O.C; 3 Division of Infectious Diseases, Department of Internal Medicine, National Taiwan University Hospital, Taipei, Taiwan, R.O.C

## Abstract

**Background:**

Progressive cell-mediated immunodeficiency with decrease of CD4+ lymphocyte count to less than or equal to 200 cells/mm^3 ^is a major risk factor for colonization with *Candida *species and development of candidiasis. Oropharyngeal candidiasis may occur in up to 90% of human immunodeficiency virus (HIV)-infected patients during the course of the disease. This study is to determine the effect of prolonged highly active antiretroviral therapy (HAART) on oropharyngeal colonization with *Candida *species and oral candidiasis.

**Methods:**

A prospective, longitudinal follow-up study in HIV-infected patients receiving HAART.

**Results:**

The mean CD4+ count increased from 232.5 to 316 cells/mm^3 ^and the proportion of patients whose CD4+ count less than 200 cells/mm^3 ^decreased from 50.0% to 28.9% (*p *= 0.0003) in patients receiving HAART for at least 2 years. The prevalence of oral candidiasis decreased from 10.6% to 2.1% (*p *= 0.004). The decrease in *Candida *colonization was less impressive, falling from 57.8% to 46.5 % (*p *= 0.06). Of the 142 patients enrolled in at least two surveys, 48 (33.8%) remained colonized with *Candida *and 42 (29.6%) remained negative. In the remaining 52 patients, 34 switched from culture positive to negative, and an increase in CD4+ lymphocytes was noted in 91.2% of them. Among the 18 patients who switched from culture negative to positive, 61.1% also demonstrated an increase in CD4+ lymphocyte count (*p *= 0.01).

**Conclusion:**

These findings indicate that HAART is highly effective in decreasing oral candidiasis in association with a rise in CD4+ lymphocyte counts, but only marginally effective in eliminating *Candida *from the oropharynx.

## Background

Mucosal candidiasis, including oropharyngeal, esophageal, and vaginal candidiasis, is common among human immunodeficiency virus (HIV)-infected patients [2,7]. Oropharyngeal candidiasis may occur in up to 90% of HIV-infected patients during the course of the disease [9]. Progressive cell-mediated immunodeficiency with decrease of CD4+ lymphocyte count to ≤ 200 cells/mm^3 ^is a major risk factor for colonization with *Candida *species and development of candidiasis [1,3].

The introduction of highly active antiretroviral therapy (HAART) has led to a marked decrease in mortality and morbidity [6,8] as well as the incidence of opportunistic infections among HIV-infected patients [4]. It is less clear whether immune reconstitution protects HIV-infected patients from colonization with *Candida *species. These three prospective longitudinal follow-up surveys (in 1999, 2001, and 2002) were designed to determine the effect of prolonged HAART on oropharyngeal candidiasis and colonization with *Candida *species in Taiwan.

## Methods

HIV-infected patients followed regularly at the acquired immune deficiency syndrome (AIDS) clinic at the National Taiwan University Hospital (NTUH) were enrolled in the study. The patients underwent three rounds of surveys for oral candidiasis in 1999, 2001, and 2002. A standardized case form was used to retrieve demographic information, CD4+ lymphocyte counts obtained from dates nearest to the survey culture dates and viral load. Hospitalization and receipt of antibiotics and anti-fungal drugs within three months of sampling were also recorded. For patients participated in all three surveys, the clinical data of 1999 and 2002 were used for analysis. Inclusion criteria consisted of continuous receipt of HAART, at least two complete clinical and laboratory observations during the study period and verbal informed consent. The institutional review board of the NTUH has approved the study.

Cultures of the oropharyngeal and tonsillar regions were performed using a dry sponge swab (EZ Culturette, Becton Dickinson, Sparks, MD) as described previously [3,5]. The swabs collected in 1999 were plated on Sabouraud Dextrose Agar (SDA) medium with chloramphenicol and gentamicin (BBL), and those collected in 2001 and 2002 were plated on Chromagar Candida (BBL). All cultures were incubated at 30°C. For diagnosis, the isolates were first tested by the germ tube assay. The VITEK Yeast Biochemical Card (YBC, bioMerieux, St. Louis, MI, USA) was used to identify isolates that failed to form germ tubes. The API-32C (Marcy, L'ETOILE, France) was used to assess the results when the VITEK-YBC showed less than 90% confidence.

Plasma viral RNA load (PVL) and CD4+ lymphocyte count were usually determined at the baseline at the diagnosis of HIV infection and/or immediately before initiation of antiretroviral therapy. In those who did not initiate antiretroviral therapy, those tests were conducted on 4–6 month intervals; in those who did initiate the antiretroviral therapy, those tests were first conducted at 1 month after the beginning of the therapy, and then onto 4-month intervals regularly. When the therapy is to be changed due to virologic failure, testing protocol would be the same as those who initiated therapy for the first time. PVL were quantified using reverse transcriptase-polymerase chain reaction (RT-PCR, Roche Amplicor, version 1.5) with a detection limit of 400 copies/ml and CD4+ counts were determined using FACFlow (Becton Dickinson). Oropharyngeal candidiasis was characterized by painless, creamy white, plaque-like lesions on the buccal or oropharyngeal mucosa or tongue surface.

All clinical and laboratory data were entered into a relational database designed for Microsoft Access 97 (Microsoft, Redland, WA). The statistical significances of the differences in frequencies and proportions were determined by the Chi-square test with Mantel-Haenszel correction.

## Results

During this four-year-long study, there were 142 patients met the inclusion criteria. The mean age for them at the initial survey was 38.8 years and there were 93.7% males. Of these patients, 41 received the first HAART when they enrolled in this study. Among the remaining, 52, 31, 9, 8, and 1 patients have participated the HAART program for 1, 2, 3, 4, and 7 years, respectively, before they enrolled in this study. There were 11 patients participating only in the 1999 and 2001 surveys (group 1), 83 patients only in the 2001 and 2002 surveys (group 2), and 48 patients in all three surveys. Upon follow-up, the mean CD4+ lymphocyte count rose from 232.5 ± 180.1 cells/mm^3 ^to 316 ± 197.0 cells/mm^3^. The proportion of patients whose CD4+ count was ≤ 200 cells/mm^3 ^decreased from 50.0% to 28.9 % (p = 0.0003) (Table 1). There was also a significant decrease between the initial and follow-up surveys in the percentage of patients who were hospitalized or received antifungal drugs and antibiotics (Table 1). These findings were independent of age and gender.

**Table 1:** Characteristics of 142 patients receiving HAART

	First survey				Subsequent survey				
			
Group	1 (N = 11)	2 (N = 83)	3 (N = 48)	Total	1 (N = 11)	2 (N = 83)	3 (N = 48)	Total	p
Characteristic	number of isolates (percentage)				number of isolates (percentage)				
CD4+ count > 200	5 (45.5)	42 (50.6)	24 (50)	71 (50)	10 (10.9)	55 (66.3)	36 (75)	101 (71.1)	0.0003
CD4+ count ≤ 200	6 (54.5)	41 (49.4)	24 (50)	71 (50)	1 (9.1)	28 (33.7)	12 (25)	41 (18.9)	0.0003
Candida culture positive	7 (63.3)	48 (57.8)	27 (56.3)	82 (57.7)	6 (54.5)	37 (44.6)	23 (47.9)	66 (46.5)	0.06
Oral candidiasis	1 (9.1)	10 (12)	4 (8.3)	15 (10.6)	0	2 (2.4)	1 (2.1)	3 (2.1)	0.004
Hospitalized*	1 (9.1)	15 (18.1)	10 (20.8)	26 (18.3)	1 (9.1)	4 (4.8)	1 (2.1)	6 (4.2)	0.0002
Received anti-fungal drugs*	2 (18.2)	10 (12)	7 (14.6)	19 (13.4)	0	3 (3.6)	1 (2.1)	4 (2.8)	0.002
Received antibiotics*	9 (81.8)	29 (34.9)	32 (66.7)	70 (49.3)	2 (18.2)	16 (19.3)	4 (8.3)	22 (15.5)	< 0.0001
Plasma viral load (> 50 copies/ml) (N = 82)	ND	59 (72)	ND	ND	ND	22 (26.8)	ND	ND	< 0.0001

* Within three months of the Candida culture, p value is calculated by total number of isolates

Group 1, patients only in 1999 and 2001; group 2, patients only in 2001 and 2002; and group 3, patients in all three years.

In all, the prevalence of oral candidiasis decreased from 10.6 to 2.1 % (*p *= 0.004), while the change in prevalence of *Candida *colonization was less impressive, falling from 57.8% to 46.5 % (*p *= 0.06). Among the patients, 48 (33.8%) remained colonized with *Candida *and 42 (29.6%) remained negative. And in the remaining 52 patients, 34 switched from culture positive to negative alone with an increase in CD4+ cells in 91.2% of them. Among the 18 patients switching from culture negative to positive, 61.1% also demonstrated an increase in CD4+ counts. In the total 284 samplings, there were 65 with CD4+ counts ≤ 200 and were also culture-positive for *Candida *whereas there were 47 with CD4+ counts ≤ 200 but were culture-negative (*p *= 0.11). Thus, in addition to the CD4+ count, some other factors appear to have significant effects on oral colonization by *Candida*.

## Discussion

We have previously shown that hospitalization and receipt of antibiotics were risk factors for oropharyngeal colonization by *Candida *and that a low CD4+ lymphocyte count (≤ 200 cells/mm^3^) was a risk factor for developing oral candidiasis [3]. It is well known that HIV-infected patients are more likely to be colonized by *Candida *than healthy individuals [3,7]. Our current findings are consistent with previous reports. The dramatic reduction in oral candidiasis found in this study is also consistent with reports that HAART markedly decreases mortality and morbidity as well as the incidence of AIDS-defining opportunistic infections [6,8]. It was therefore somewhat surprising to find that the frequency of oropharyngeal colonization by *Candida*, although close to significant (*p *= 0.06), was not as dramatically decreased. Possible explanations include poor compliance, failure to reduce the viral load and raise the CD4+ lymphocyte count or dissociation between colonization and infection. The most likely scenario is that most patients receiving HAART continue to be colonized by *Candida*, but do not develop oral candidiasis.

## Conclusion

Increase of the CD4+ lymphocyte counts is required but not sufficient to protect HIV-infected patients from colonization by *Candida*. Further studies are needed to better define the relationship between *Candida *colonization and infection in patients with AIDS.

## Abbreviations used

HIV, human immunodeficiency virus; HAART, highly active antiretroviral therapy; AIDS, acquired immune deficiency syndrome; NYUH, National Taiwan University Hospital; PVL, Plasma viral RNA load; RT-PCR, reverse transcriptase-polymerase chain reaction

## Competing interests

The author(s) declared that they have no competing interests.

## Authors' contributions

YLY designed the study with contribution with HJL and CCH. YLY drafted the manuscript. HJL conducted the experiments with contribution from YL. CCH collected swabs and patients' information.

## Pre-publication history

The pre-publication history for this paper can be accessed here:


